# NUCB2/Nesfatin-1 drives breast cancer metastasis through the up-regulation of cholesterol synthesis via the mTORC1 pathway

**DOI:** 10.1186/s12967-023-04236-x

**Published:** 2023-06-05

**Authors:** Siyi Ning, Caiying Liu, Kangtao Wang, Yubo Cai, Zhicheng Ning, Ming Li, Liang Zeng

**Affiliations:** 1 Department of Immunology, College of Basic Medical Sciences, Central South University, Changsha, 410028 China; 2 Department of General Surgery, The Xiangya Hospital, Central South University, Changsha, 410028 China; 3 Hunan Normal University School of Medicine, Changsha, 410031 China; 4 Department of Pathology, Guangzhou Women and Children’s Medical Center, Guangzhou Medical University, Guangdong Provincial Clinical Research Center for Child Health, National Children’s Medical Center for South Central Region, Guangzhou, 510623 China

**Keywords:** Breast cancer, Cholesterol synthesis, Metastasis, Nucleobindin-2/Nesfatin-1

## Abstract

**Background:**

Reprogramming lipid metabolism for tumor metastasis is essential in breast cancer, and NUCB2/Nesfatin-1 plays a crucial role in regulating energy metabolism. Its high expression is associated with poor prognosis in breast cancer. Here, we studied whether NUCB2/Nesfatin-1 promotes breast cancer metastasis through reprogramming cholesterol metabolism.

**Methods:**

ELISA was employed to measure the concentration of Nesfatin-1 in the serum of breast cancer patients and the control group. Database analysis suggested that NUCB2/Nesfatin-1 might be acetylated in breast cancer, which was confirmed by treating the breast cancer cells with acetyltransferase inhibitors. Transwell migration and Matrigel invasion assays were conducted, and nude mouse lung metastasis models were established to examine the effect of NUCB2/Nesfatin-1 on breast cancer metastasis in vitro and in vivo. The Affymetrix gene expression chip results were analyzed using IPA software to identify the critical pathway induced by NUCB2/Nesfatin-1. We evaluated the effect of NUCB2/Nesfatin-1 on cholesterol biosynthesis through the mTORC1-SREBP2-HMGCR axis by utilizing mTORC1 inhibitor and rescue experiments.

**Results:**

NUCB2/Nesfatin-1 was found to be overexpressed in the breast cancer patients, and its overexpression was positively correlated with poor prognosis. NUCB2 was potentially acetylated, leading to high expression in breast cancer. NUCB2/Nesfatin-1 promoted metastasis in vitro and in vivo, while Nesfatin-1 rescued impaired cell metastasis induced by NUCB2 depletion. Mechanistically, NUCB2/Nesfatin-1 upregulated cholesterol synthesis via the mTORC1 signal pathway, contributing to breast cancer migration and metastasis.

**Conclusions:**

Our findings demonstrate that the NUCB2/Nesfatin-1/mTORC1/SREBP2 signal pathway is critical in regulating cholesterol synthesis, essential for breast cancer metastasis. Thus, NUCB2/Nesfatin-1 might be utilized as a diagnostic tool and also used in cancer therapy for breast cancer in the future.

**Supplementary Information:**

The online version contains supplementary material available at 10.1186/s12967-023-04236-x.

## Introduction

In recent years, an increasing number of studies have identified lifestyle as the primary determinant of the incidence and development of breast cancer. Among lifestyle factors, obesity [1], metabolic syndrome [2], type II diabetes [3], and hypercholesterolemia have all been recognized as risk factors for breast cancer. Experimental evidence has linked dysregulated cholesterol homeostasis to cancer pathobiology, with excessive accumulation of cholesterol playing a critical role in the progression of breast cancer and being closely associated with poorer outcomes [4, 5].

Cholesterol serves as a precursor of steroid hormones, bile acids, and vitamin D, and is an essential structural component of cell membranes enriched in membrane lipid rafts that transmit signals across the membrane [6]. Thus, cholesterol likely affects cellular architecture and signal transduction. In 1953, Waxler et al. reported that breast cancer incidence increased in murine models with elevated cholesterol levels [7]. Since then, increasing evidence has indicated that elevated cholesterol is a risk factor for both the onset and recurrence of breast cancer [8]. Cholesterol is an essential component of lipid rafts, which transduce PI3K/Akt signaling, promoting breast cancer proliferation and pulmonary metastasis [9]. Additionally, 27-hydroxycholesterol (27HC), a primary metabolite of cholesterol, can act as a ligand for both estrogen receptors (ERs) and liver X receptors (LXRs). Activation of ER by 27HC induces cellular proliferation in breast cancer cells [10]. Conversely, LXRs, which can decrease cellular cholesterol uptake and increase efflux, induce epithelial-to-mesenchymal transition (EMT) and subsequent breast cancer metastasis [10].

Cholesterol biosynthesis is controlled by sterol regulatory element-binding protein 2 (SREBP2), a key transcription factor in cholesterol biosynthesis, and its downstream genes, including rate-limiting enzymes such as 3-hydroxy-3-methyl glutaryl-CoA reductase (HMGCR), 3-hydroxy-3-methyl glutaryl-CoA synthase 1 (HMGCS), and squalene synthase (SS) [11]. It has been demonstrated that the mammalian target of rapamycin complex 1 (mTORC1) regulates cholesterol biosynthesis by controlling the transcriptional factor SREBP2. Abnormal activation of the mTOR signaling pathway is present in 70% of breast cancer [12–14]. Overexpression of mTOR is associated with poor prognosis of breast cancer, and phosphorylated mTOR is highly expressed in developed breast cancer[15, 16]. Therefore, further exploration of the regulatory mechanism of mTOR signaling pathway can facilitate to search for potential anti-tumor targets.

Human Nucleobindin-2 (NUCB2), first described in 1994 and extensively studied since 2006 [17, 18], is a DNA/Ca^2+^ binding protein with metabolic functions that include food intake, energy metabolism, and regulation of the immune, cardiovascular, and endocrine systems [18, 19]. The NUCB2 gene encodes a 396 amino acid-long precursor peptide and a 24 amino acid-long signal peptide. NUCB2 can be proteolytically converted into peptide segments: Nesfatin-1, Nesfatin-2, and Nesfatin-3. Oh et al. found that intraventricular injection of NUCB2 could reduce mice's nocturnal feeding and weight gain, and identified Nesfatin-1 as an influential fragment. They also found that NUCB2/Nesfatin-1 is a secretory component of various body fluids, including saliva, synovial fluid, and serum [18, 19]. As NUCB2 and Nesfatin-1 are co-localized, these two names are used interchangeably. It was reported that Nesfatin-1 is a novel depot-specific adipokine preferentially produced by subcutaneous tissue [20]. Interestingly, more and more studies have shown that NUCB2 is an oncogene that improves tumor invasion and metastasis [21, 22]. Patients with high NUCB2/Nesfatin-1 expression have significantly poor overall survival and increased recurrence rates in breast cancer [21, 23]. Therefore, NUCB2/Nesfatin-1 is suggested to be a new prognostic and predictive marker in breast cancer.

In our previous study, we utilized Isobaric Tags for Relative and Absolute Quantitation (iTRAQ) combined with Liquid Chromatography-Tandem Mass Spectrometry (LC–MS)/MS to identify differentially expressed proteins involved in breast cancer metastasis [24]. Among the proteins identified, NUCB2 was found to be highly expressed in paired primary breast tumors and metastatic lymph nodes, and was positively correlated with poor survival of breast cancer patients [24]. Other research groups have also observed a significant positive correlation between NUCB2 expression, nodal metastasis, and clinical stage [21]. However, the underlying molecular mechanism behind NUCB2's effect is not yet fully understood.

Interestingly, increasing evidence suggests that the significant signal pathway induced by NUCB2 is associated with mTOR. For example, NUCB2 has been shown to regulate glucose intake, liver insulin sensitivity, and glucose tolerance through AMPK/mTOR and mTOR-STAT3 signaling pathway [25, 26]. In addition, Kan et al. found that NUCB2 regulates ZEB1 expression via the liver kinase B1(LKB1)/AMPK/mTOR pathway, resulting in mesenchymal properties in colorectal cancer [27]. Furthermore, studies have reported that NUCB2 enhances the epithelial-mesenchymal transition in renal cell carcinoma [28].

In this study, we found the major signal pathway involved by NUCB2 is cholesterol synthesis assayed by GeneChip detection combined with Ingenuity Pathway Analysis (IPA) analysis. Thus we intended to explore whether NUCB2 regulates SREBP2 expression through mTORC1, mediating cholesterol synthesis and leading to malignant phenotype in breast cancer.

Post-transcriptional modifications (PTMs) refer to chemical modifications that occur on a protein after its translation [29, 30]. There is increasing evidence that oncogene activation and/or tumor suppressor gene inactivation can be regulated by diverse PTMs [31, 32]. Protein acetylation is a reversible and evolutionarily conserved PTM that is regulated by the opposing actions of lysine (K) acetyltransferases (KATs) and histone deacetylases (HDACs). Although hundreds of acetylation sites have been identified in human breast cancer [33], the exact molecular mechanisms underlying the effects of this abnormal PTM on various pathophysiological processes in breast cancer are only beginning to be investigated. To preliminarily explore the mechanism of high expression of NUCB2 in breast cancer, we analyzed the potential acetylation sites in NUCB2 and the acetyltransferases involved using the CUCKOO database. We found that lysine at multiple locations of NUCB2 may be acetylated. Therefore, we treated breast cancer cell lines with tool enzymes to detect this potential PTM. The results suggested that NUCB2 may be overexpressed in breast cancer cells potentially through protein acetylation.

In all, our findings suggest that NUCB2/Nesfatin-1 regulates cholesterol synthesis by improving SREBP2 and HMGCR abundance via the mTORC1 pathway, resulting in high metastasis ability in breast cancer both in vitro and in vivo. Our results provide valuable insight into NUCB2/Nesfatin-1 as a regulator of cholesterol synthesis and a potential drug target for breast cancer therapy. Furthermore, these findings have significant implications for the development of prognostic markers in breast cancer.

## Materials and methods

### Collection of clinical samples and detection of Nesfatin-1 concentration by ELISA

Serum samples were collected from 40 breast cancer patients and 40 healthy donors at the Hunan Province Cancer Hospital. The serum was isolated by centrifugation at 1000 *g* for 10 min at room temperature and stored at – 70 ºC until ELISA was performed. The study was approved by the Ethics Committee at the Hunan Province Cancer Hospital (Hunan, China,; permission number: 2013-40), and written informed consent was obtained from all participants. The concentration of Nesfatin-1 in the serum was measured using the human Nesfatin-1/Nucleobindin-2 ELISA kit (BOSTER, #EK1138) according to the manufacturer's instructions.

### Cell lines

Breast cancer cell lines BT-549, MDA-MB-231, MDA-MB-468, and MCF-7 as well as an immortalized mammary epithelial-like cell line, MCF-10A were obtained from Procell biological company (Shanghai, China). BT-549 and MCF-7 cells were cultured in RPMI-1640 (Procell) supplemented with 10% (v/v) fetal bovine serum (FBS), 0.01 mg/mL insulin (Procell), 2 mM l-glutamine, 0.1 mg/mL streptomycin, and 100 U/mL penicillin at 37 °C in a humidified atmosphere containing 5% CO_2_. MDA-MB-231 and MDA-MB-468 cells were cultured in Leibovitz's L-15 (Gibco) with 10% (v/v) FBS, 2 mM l-glutamine, 0.1 mg/mL streptomycin, and 100 U/mL penicillin at 37 °C in a standard humidity incubator. MCF-10A was cultured in DMEM (Procell) supplemented with 5% horse serum, 20 ng/mL epidermal growth factor, 0.5 μg/mL hydrocortisone, 0.1 mg/mL streptomycin, 100 U/mL penicillin, and 0.01 mg/mL insulin at 37 °C in a humified atmosphere containing 5% CO_2_.

### Antibodies and reagents

The antibodies used included anti-GAPDH (Bioword, AP0063), anti-NUCB2 (Proteintech, 26712-1-AP), anti-E-cadherin (Cell Signaling Technology, 24E10), anti-N-cadherin (Cell Signaling Technology, D4R1H), anti-Vimentin (Cell Signaling Technology, D21H3), anti-HisG (Cell Signaling Technology, D3I1O), anti-HMGCR (Abcam, ab242315), anti-SREBP2 (Abcam, ab30682), horseradish peroxidase (HRP) labeled sheep anti-Rabbit IgG (Cell Signaling Technology, 7074P2), and HRP labeled sheep anti-Mouse IgG-HRP (Sigma, A9917). The primary antibody titers for Western Blot or Immunohistochemistry are listed in Additional file 2: Table S1. Recombinant human purified Nesfatin-1 was purchased from Peprotech Company, and Rapamycin was obtained from Selleck Chemicals. Cholesterol was purchased from Beyotime, China.

### Cell transfection

The lentiviral-based small hairpin RNA (shRNA) targeting NUCB2 was constructed by Genchem Biotechnology Co. Ltd., Shanghai. They also provided the control lentivirus with scrambled shRNA. The target sequence of NUCB2 was 5′-CAGATAAACACTTCAGAGAAA-3′, and the scramble sequence was 5′-TTCTCCGAACGTGTCACGT-3′. MDA-MB-231 and BT-549 cells were infected with the lentivirus particles and maintained in L-15 or 1640 containing 2 μg/mL or 1 μg/mL puromycin, respectively. After 2 weeks of selection, reverse transcription-quantitative polymerase chain reaction (RT-qPCR), Western blot, and ELISA were performed to detect NUCB2/Nesfatin-1 expression and confirm the stable cell lines. The cDNA of HMGCR was subcloned into the pcDNA3.1–6 × HisG plasmid. For transient transfection, 2 × 10^5^ cells were seeded into a 6-well plate and cultured overnight. The cells were transfected with 2.5 μg pcDNA3.1–6 × HisG-HMGCR plasmid or empty vector DNA using Lipofectamine 3000 (Thermofish, L3000015) according to the manufacturer's protocol. After 48 h, the cells were harvested for Western blot, migration, and invasion experiments.

### Publicly available clinical data analysis

The Cancer Genome Atlas (TCGA) gene expression data for 1097 breast cancer patients and 114 controls were obtained from UALCAN [34]. Online tools were used to show the expression difference of NUCB2 between normal breast tissue and breast cancer. The Kaplan–Meier online analysis database shows the survival data associated with differential expression of NUCB2. The expression relationship between NUCB2 and SREBP2/HMGCR was analyzed through the Xena website [35].

### RNA extraction and reverse transcription-quantitative polymerase chain reaction (RT-qPCR)

Total RNA was extracted from cultured cells using TRIzol reagents (Life Technologies) and reverse-transcribed to cDNA using a kit (TaKaRa, RR047A). Gene expression was analyzed using Fast SYBR Green Master Mix (TaKaRa, RR820A) on the CFX96 Real-Time System (Bio-Rad). The primers for qPCR are listed in Additional file 2: Table S2. The relative expression of each gene was calculated according to the 2^−∆∆Ct^ method. The results were normalized to GAPDH for measuring the relative mRNA expression. At least triplicate experiments were performed in each sample.

### Western blot (WB) analysis

The Western blot analysis was adapted from Chun Zou [36]. The antibodies used are listed in Additional file 2: Table S1. Briefly, cells were lysed in RIPA buffer with a protease inhibitor cocktail (Selleck). Thirty micrograms of protein were denatured by boiling at 95 ℃ for 5 min and resolved by sodium dodecyl sulfate–polyacrylamide gel (SDS-PAGE) electrophoresis. The proteins were then transferred to a PVDF membrane, followed by blocking with 5% fat-free milk in TBS buffer with 0.1% Tween-20 (TBS-T) at room temperature for 1 h. The membranes were sliced according to the molecular weights, incubated with primary antibodies at 4 °C overnight, washed with TBS-T, and incubated with HRP-conjugated secondary antibodies for 2 h at room temperature. Signals were detected by an enhanced chemiluminescence (ECL) system (Amersham Pharmacia Biotech, Arlington Heights, IL). Each blot was repeated three times. GAPDH was used as the loading control. Relative quantitative was performed by measuring the intensity of the strips with Image J.

### Immunohistochemistry (IHC) Staining

Tissue samples of the nude mice were preserved through paraffin embedding and mounted onto 3-aminopropyltrioxysilane-coated slides, dewaxed, and hydrated. The IHC protocol was described previously[24]. Briefly, antigen retrieval was performed in citric acid retrieval solution, heated in a microwave, and then cooled at room temperature. Endogenous peroxidase activity was quenched by 3% H_2_O_2_ for 15 min. After blocking nonspecific binding, the slides were incubated overnight with the primary antibody at 4ºC. The slides were incubated with biotinylated secondary antibody followed by incubating with the streptavidin-peroxidase conjugate and color development using the DAB/H_2_O_2_ system.

### Transwell migration and matrigel invasion assays

Transwell migration and Matrigel invasion assays were performed in 24-well culture plates with inserts of 8 μm pore membranes (Falcon) pre-coated without or with Matrigel (Corning). 4 × 10^4^ cells in 0.2% bovine serum albumin (BSA) were seeded in the upper chamber. The lower chamber was filled with 500 μL medium supplemented with 10% FBS. Twenty-four hours later, the cells on the bottom surface of the upper chamber were fixed with 4% paraformaldehyde for 20 min and stained with 0.1% Crystal Violet. The migrated or invaded cells were counted and photographed under a microscope. Five fields per membrane were counted in each group. All experiments were performed twice, at least.

### Antibody-blocking assay

To verify whether NUCB2 exerts biological effects through Nesfatin-1, we used an antibody-blocking test. Briefly, breast cancer cells (2 × 10^5^) were seeded into a six-well plate and cultured overnight. Rabbit anti-human Nesfatin-1 antibody (1.4 μg/mL) or rabbit IgG (1.4 μg/mL) were added into the wells, respectively. After incubation for 12 h, cells were conducted by migration and invasion experiments.

### Cell proliferation and colony formation assays

To investigate the potential cytotoxicity of Nesfatin-1, a cell proliferation assay was performed. Specifically, 2 × 10^4^ cells in 100 μl of medium were seeded per well in a 96-well plate and exposed to different concentrations of Nesfatin-1 (0, 100, 1,000, 10,000 pg/mL). After two days, a CCK-8 assay was conducted to evaluate cell viability. In this assay, viable cells were incubated with diluted CCK-8 reagents (Dojindo Molecular Technologies, Gaithersburg, MD, USA) for 2 h, and the absorbance at a wavelength of 450 nm was measured to determine the viable cell count in each well.

For the colony formation assay, 1000 cells were plated in triplicate in a six-well plate, cultured for 2–3 weeks, and refreshed with medium every three days. At the end of the incubation period, the cells were fixed with 1% paraformaldehyde for 20 min and stained with 0.1% crystal violet for 10 min. The number of cell colonies was then counted.

### Cellular cholesterol determination assay

To detect the cholesterol concentration in cells, the total cellular cholesterol detection kit (Applygen, E1015) was utilized. Briefly, the cells were washed twice with phosphate buffer (PBS), lysed with lysis buffer, and scraped off. The lysates were then centrifuged at 12,000*g* for 10 min, and 10 μL of the supernatant was transferred into a 96-well plate. Next, the samples were incubated with 190 μL of cholesterol detection working solution at 37 ℃ for 20 min. Finally, the absorbance at a wavelength of 562 nm was measured, and the cholesterol concentration was calculated using a standard curve. The lysate concentration was also measured using a BCA protein quantitative kit (Thermo scientific, #23227) to calculate the cells' cholesterol content per microgram of protein.

### Ingenuity pathway analysis (IPA)

To identify downstream genes regulated by NUCB2, the Affymetrix gene expression chip (Path Array) was used to measure gene expression in shCTRL-MDA-MB-231 and shNUCB2-MDA-MB-231 cell lines. Total RNA was extracted from the cell lines and reverse-transcribed into cDNA, which was used as the template to synthesize complementary RNAs (cRNAs) with the GeneChip IVT Labeling Kit. The cRNAs were then fragmented, and an appropriate hybridization solution was added. After elution, the chip was scanned, and IPA analysis was performed using GCOS software.

### In vivo assays

The animal experiments were approved by the Ethics Committee of Genechem Company (Shanghai, China; permission number: GSZE0155048), conforming to internationally accepted principles in the care and use of experimental animals (NRC, 2011). Twenty female Balb/c nude mice (4 weeks old) were randomly divided into two groups with ten mice in each group. For the lung metastasis model, shNUCB2-MDA-MB-231 or shCTRL-MDA-MB-231 stable cell lines (2 × 10^6^ cells/mouse/100 μL) were injected into the tail vein of each nude mouse. D-luciferin (10 μL/g) was regularly injected intraperitoneally for later observation. The animals were sacrificed in the 12th week after injection, and their lungs were dissected, embedded in paraffin, and counted under a microscope.

### Statistical analysis

Statistical analysis and visualization of all results in this study were performed using GraphPad Prism 6.01, IBM SPSS 20.0, ImageJ, and Adobe Illustrator cc 2018 software. All P-values of statistical data are based on bilateral statistical tests, and data with *P* < 0.05 are considered statistically significant. #: no significance (ns), *P* > 0.05, *P < 0.05, **P < 0.01, and ***P < 0.001.

## Results

### NUCB2/Nesfatin-1 is highly expressed in breast cancer and correlates with poor prognosis

**Fig. 1:**
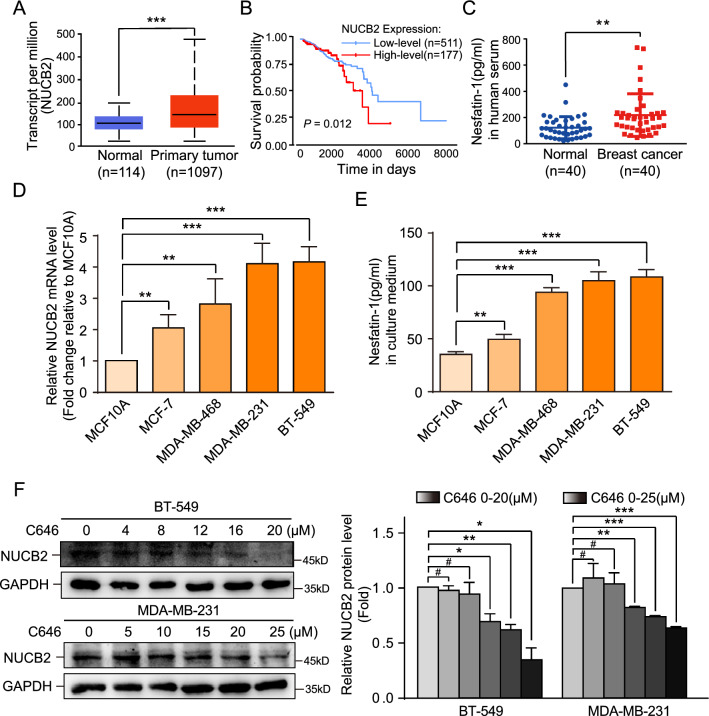
NUCB2/Nesfatin-1 is overexpressed in breast cancer and correlates with poor prognosis. **A** NUCB2 mRNA expression in primary breast cancer tissues (n = 1097) and normal tissues (n = 114) as analyzed by TCGA data. **B** Kaplan–Meier survival curve based on univariate NUCB2 level using TCGA data. **C** ELISA results of Nesfatin-1 concentration in serum samples of healthy donors (n = 40) and breast cancer patients (n = 40). Each dot represents the mean Nesfatin-1 concentration in triplicates of each sample. **D** RT-qPCR analysis of NUCB2 mRNA expression in immortalized mammary epithelial-like cell line and four breast cancer cell lines. **E** ELISA results of Nesfatin-1 concentration in supernatants from immortalized mammary epithelial-like cell line and four breast cancer cell lines. 2 × 10^5^ breast cancer cells were seeded in a six-well plate containing 2 mL medium and cultured overnight. The supernatants were collected and Nesfatin-1 concentration was detected by ELISA. **F** Representative Western blots and relative quantitative data of NUCB2 expression in BT-549 and MDA-MB-231 cells treated with P300/CREBBP inhibitor C646. Representative results from at least three independent experiments are shown. Statistical significance was determined using unpaired T-test. #*P* > 0.05, *P < 0.05, **P < 0.01, ***P < 0.001

In our previous study, we highlighted the crucial role of NUCB2 in breast cancer regulation, particularly in lymph node metastasis [24]. Furthermore, we demonstrated that the positive expression of NUCB2 in primary breast tumors is an independent risk factor for the poor prognosis of breast cancer patients [24]. In this study, we further investigated the expression of NUCB2 in breast cancer by analyzing the TCGA database and found that NUCB2 expression was significantly higher in breast cancer tissues (n = 1097) than in normal tissues (n = 114) (*P* < 0.001, Fig. 1A). Our Kaplan–Meier survival curve analysis also revealed that high-level expression of NUCB2 was associated with poor survival (*P* < 0.05, Fig. 1B).

To investigate the clinical significance of NUCB2, we collected serum samples from 40 healthy donors and 40 breast cancer patients and measured the concentration of Nesfatin-1, a cleavage product of NUCB2 (Additional file 1: Fig. S1A), using ELISA. Our results showed that the average concentration of Nesfatin-1 in breast cancer patients was significantly higher than that in healthy controls (*P* < 0.01, Fig. 1C), which was consistent with the high expression of NUCB2 in breast cancer tissues. We also measured the levels of NUCB2/Nesfatin-1 in several breast cancer cell lines and an immortalized human mammary cell line, and found that both mRNA and protein were overexpressed in parallel in MDA-MB-231, BT-549, MDA-MB-468, and MCF-7 compared with MCF-10A, the non-cancer mammary epithelial cell line (Fig. 1D, E). Above findings suggest that Nesfatin-1 is the potentially crucial component for NUCB2 to exert its biological effects in breast cancer.

To explore the mechanism of NUCB2 overexpression in breast cancer, we used the CUCKOO database to predict potential acetylated sites and the acetyltransferases involved. Our analysis revealed that eight lysine residues in the NUCB2 protein were potentially acetylated, and the acetyltransferase with a 75% probability of involvement was cAMP-response element-binding protein (CREB) binding protein (CREBBP), while lysine (k) acetyltransferase 2B (KAT2B) had a 25% probability of involvement (Additional file 1: Fig. S1B, C). We then treated BT-549 and MDA-MB-231 breast cancer cell lines with C646, a CREBBP inhibitor, and found that the protein level of NUCB2 decreased in a dose-dependent manner with increasing concentrations of C646 (Fig. 1F. Additional file 1: Fig. S1D). These results suggest that NUCB2 may achieve high expression in breast cancer cells through acetylation, a post-translational modification (PTM). Our findings suggest that NUCB2/Nesfatin-1 overexpression in breast cancer may serve as a potential biomarker for poor prognosis and tumor aggressiveness.

### NUCB2 is required for invasion and metastasis in breast cancer in vitro and in vivo

**Fig. 2:**
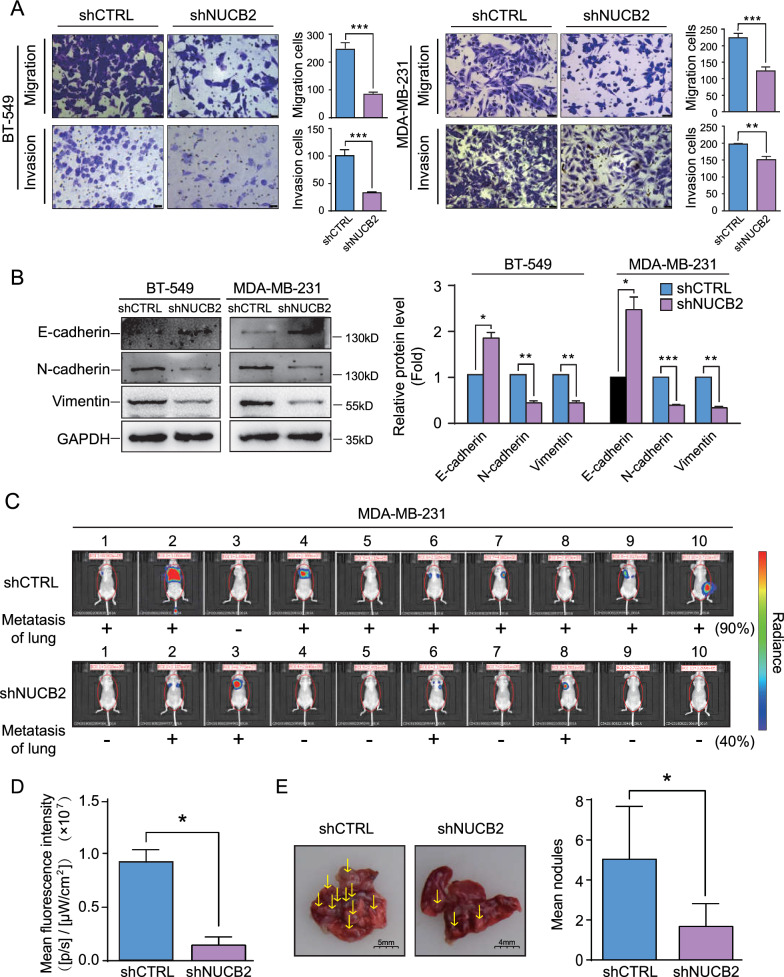
NUCB2/Nesfatin-1 is required for breast cancer invasion and metastasis in vitro and in vivo. **A** The effect of NUCB2 on cell migration and invasion in breast cancer cell lines. Briefly, 4 × 10^4^ stable cells were plated in triplicate in an upper chamber pre-coated without or with Matrigel. The lower chamber was filled with 500 μl culture medium, and cells were cultured for 24 h. The migrated or invaded cells were stained and photographed. **B** The effect of NUCB2 on the expression of EMT-related epithelial or mesothelial molecular markers detected by Western blot. Representative Western blot results and relative quantitative data are shown. **C**–**E** The effect of NUCB2 on pulmonary metastasis in the tail vein injection model. shNUCB2-MDA-MB-231 and CTRL-MDA-MB-231stable cell lines labeled by fluorescence were injected into the nude mice via vein tail. At the end of the experiment, mice were taken photos (**C**) and dissected to assess tumor nodules (**D**, **E**). The non-paired T-test was used to verify the statistical significance, *P < 0.05, **P < 0.01, ***P < 0.001

To investigate the role of NUCB2/Nesfatin-1 in breast cancer, we used shRNA to knock down NUCB2 expression in BT-549 and MDA-MB-231 cells, which express NUCB2/Nesfatin-1 at high levels (Fig. 1D, E). The efficiency of knockdown was confirmed using RT-qPCR and Western blot (Additional file 1: Fig. S2A, B), and the concentration of Nesfatin-1 in the cell culture supernatant was measured using ELISA (Additional file 1: Fig. S2C). The resulting stable cell lines were named shNUCB2-BT-549, shCTRL-BT-549, shNUCB2-MDA-MB-231, and shCTRL-MDA-MB-231. Migration and invasion assays were performed on these cell lines, and the results showed that NUCB2 downregulation impaired cell migration and invasion (*P* < 0.05, Fig. 2A). We also examined EMT-related markers in these stable cell lines and found that N-cadherin and Vimentin were decreased, while E-cadherin was increased in the shNUCB2-BT-549 and shNUCB2-MDA-MB-231 cell lines compared to the corresponding control cells (Fig. 2B). These results indicate that NUCB2 is a positive regulator of migration and invasion in breast cancer cells in vitro, likely through EMT.

To further confirm these findings, we injected shNUCB2-MDA-MB-231 or CTRL-MDA-MB-231 stable cell lines into the tail vein of 20 Balb/c nude mice (10 in each group) to establish an experimental cancer lung metastasis model. After 11 weeks, the mice were sacrificed, and the lungs were removed to evaluate lung metastasis. The results showed that the metastatic tumor rate was 40% in the shNUCB2-MDA-MB-231 group, while 90% in the CTRL-MDA-MB-231 group (Fig. 2C). Smaller lung micro-metastases were observed in the shNUCB2-MDA-MB-231 group compared to the CTRL-MDA-MB-231 group (Fig. 2C). The average fluorescence intensity and number of nodules in the shNUCB2 group were significantly lower than the control group (*P* < 0.05, Fig. 2D, E). These findings are consistent with the in vitro results, demonstrating that NUCB2 is critical in promoting breast cancer invasion and metastasis both in vitro and in vivo.

### Exogenous Nesfatin-1 recovers the phenotype caused by NUCB2 knocking down

**Fig. 3:**
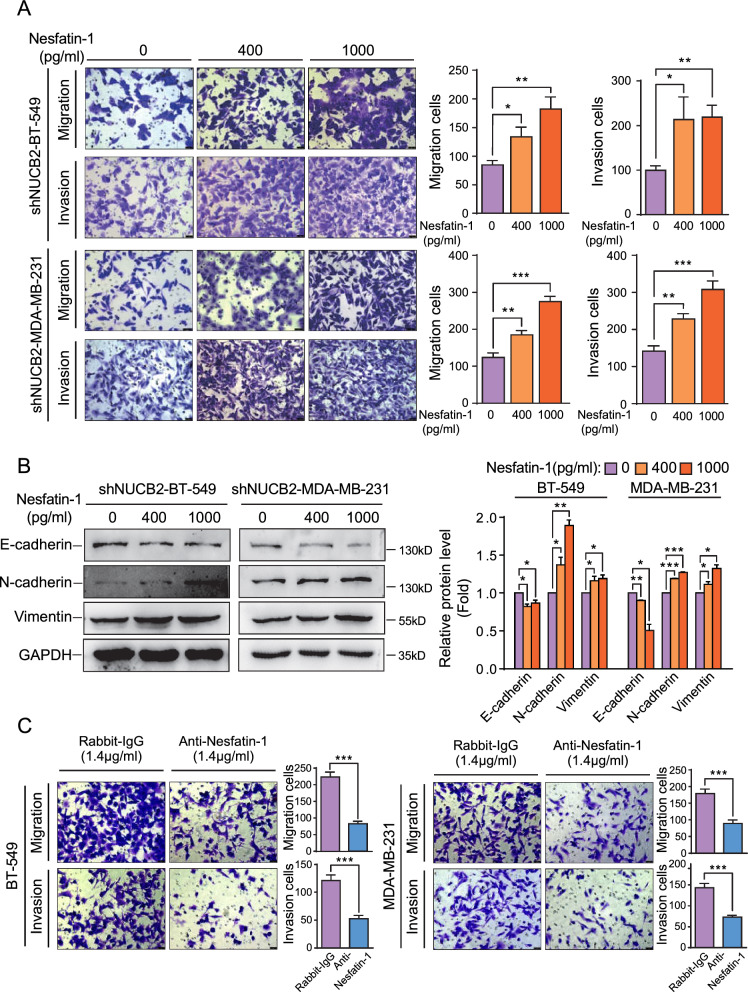
Exogenous Nesfatin-1 promotes invasion and metastasis in breast cancer. **A** The effect of different concentrations of exogenous Nesfatin-1 (0, 400, and 1000 pg/mL) on cell migration and invasion in shNUCB2-BT-549 and shNUCB2-MDA-MB-231 stable cell lines. **B** Changes in the expression of EMT-related epithelial or mesenchymal molecular markers detected by Western blot in shNUCB2-BT-549 and shNUCB2-MDA-MB-231 stable cell lines treated with Nesfatin-1 (0, 400, and 1000 pg/mL) for 48 h. Representative Western blot and relative quantitative data are shown. **C** The effect of 1.4 μg/mL Rabbit-IgG or 1.4 μg/mL rabbit anti-Nesfatin-1 antibody on cell migration and invasion in BT549 and MDA-MB-231 cell line. The statistical significance was determined using the unpaired T-test. *P < 0.05, **P < 0.01, ***P < 0.001

To further confirm the role of NUCB2 in breast cancer, we treated the breast cancer cells with exogenous Nesfatin-1 protein at doses of 400 pg/mL or 1000 pg/mL, which was shown to be less toxic (Additional file 1: Fig. S3A), and conducted migration and invasion assays on stable clones with NUCB2 knockdown. We found that exogenous Nesfatin-1 dose-dependently reversed the inhibition of cell migration and metastasis caused by NUCB2 depletion in vitro (Fig. 3A). Corresponding results were obtained by analyzing EMT-related molecular markers (Fig. 3B). Furthermore, the Nesfatin-1 antibody treatment hindered the migration and invasion in breast cancer cell lines (Fig. 3C). Nesfatin-1 also rescued the breast cancer cells' stemness in NUCB2 silenced cell lines, as demonstrated by the colony formation assay (Additional file 1: Fig. S3B). These findings suggest that Nesfatin-1 is crucial in breast cancer invasion and metastasis.

### NUCB2/Nesfatin-1 promotes tumor invasion and metastasis by activating cholesterol biosynthesis in breast cancer cells

**Fig. 4:**
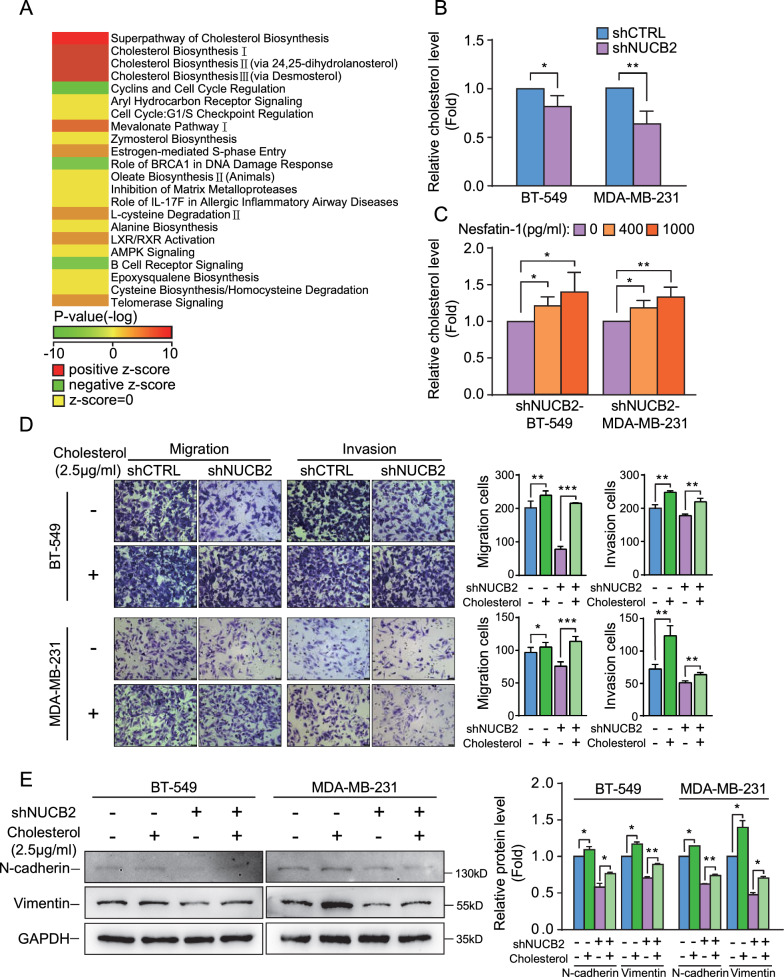
NUCB2/Nesfatin-1 promotes invasion and metastasis by activating cholesterol synthesis in breast cancer cells. **A** Classical Ingenuity Pathway Analysis (IPA) software was used to identify the pathways enriched by NUCB2. **B** Relative total cholesterol expression in two pairs of stable cell lines. **C** Relative total cholesterol expression in shNUCB2-BT-549 and shNUCB2-MDA-MB-231 stable cell lines treated with exogenous Nesfatin-1 (0, 400, or 1000 pg/mL) for 48 h. **D** Migration and invasion of two pairs of stable cell lines treated with or without 2.5 μg/mL cholesterol for 48 h. **E** Changes in EMT-related epithelial or mesothelial molecular markers detected by Western blot in two pairs of stable cell lines treated with or without 2.5 μg/mL cholesterol for 48 h. Representative Western blots and relative quantitative data are shown. Statistical significance was verified using the non-paired T-test (*P < 0.05, **P < 0.01, ***P < 0.001)

To gain insights into the signaling pathway underlying NUCB2-mediated cell invasion and metastasis, we conducted RNA sequencing analysis of NUCB2 knockdown MDA-MB-231 cells and scrambled MDA-MB-231 using the Affymetrix gene expression chip (Path-Array). Ingenuity Pathway Analysis (IPA) indicated that the cholesterol biosynthesis pathway was the most downregulated pathway in NUCB2-silenced cells, as compared to the control MDA-MB-231 cells (Fig. 4A). Further analysis revealed that the expression of five molecules involved in the cholesterol biosynthesis pathway, including farnesyl diphosphate farnesyl transferase 1 (FDFT1), 7-dehydrocholesterol reductase (DHCR7), acetyl coenzyme A acetyltransferase 2 (ACAT2), 3-hydroxy-3-methylglutaryl coenzyme A reductase (HMGCR), and 3-hydroxy-3-methylglutaryl coenzyme A synthetase 1 (HMGCS1), was significantly inhibited upon NUCB2 knockdown (Additional file 2: Table S3). Thus, we then confirmed these findings by measuring cellular cholesterol levels, which were lower in NUCB2-knockdown BT-549 and MDA-MB-231 cells than the control cells (*P* < 0.05, Fig. 4B). To further validate our results, we treated the breast cancer cells with exogenous Nesfatin-1 or vehicle and found that the lysate cholesterol concentration increased dose-dependently with Nesfatin-1 treatment (Fig. 4C). Subsequently, we treated stable cell lines with 2.5 μg/mL cholesterol and found that the decrease in invasion and metastasis in NUCB2 knockdown BT-549 and MDA-MB-231 cells was rescued by exogenous cholesterol (Fig. 4D). These findings were further supported by our analysis of EMT-related molecular markers (Fig. 4E). Taken together, our results suggest that NUCB2/Nesfatin-1 promotes the synthesis of cholesterol in breast cancer cells, thereby enhancing cell invasion and metastasis.

### NUCB2/Nesfatin-1 upregulates the expression of SREBP2 and HMGCR, critical molecules of cholesterol synthesis, by activating the mTORC1 pathway

**Fig. 5:**
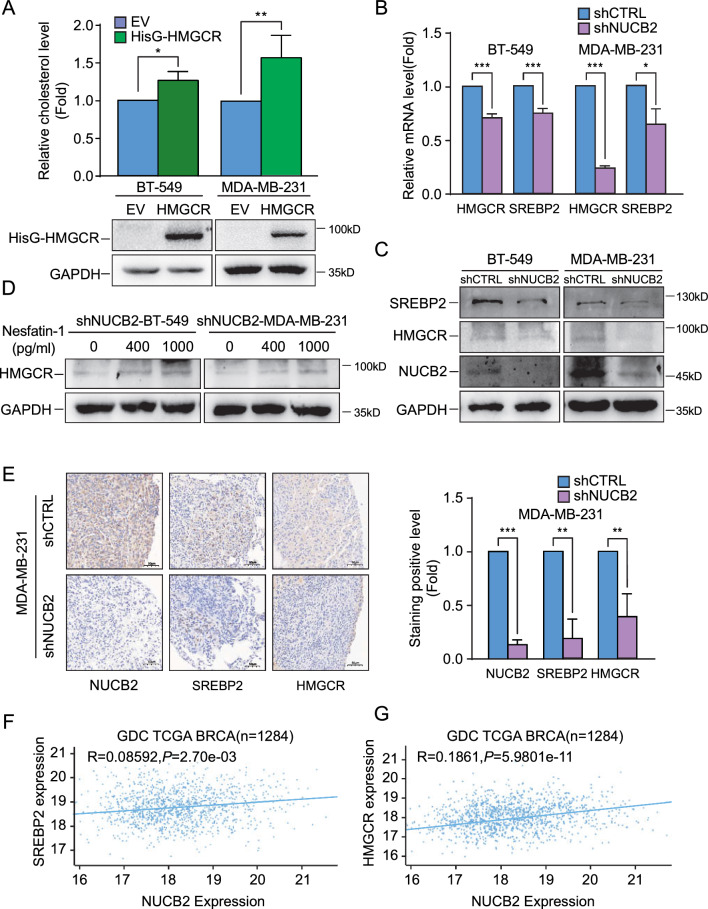
NUCB2/Nesfatin-1 induces upregulation of the SREBP2/HMGCR axis in breast cancer in vitro and in vivo. **A** Total cholesterol concentration was measured in NUCB2-silenced and scrambled BT-549 and MDA-MB-231 stable cell lines transiently transfected with either empty vector or pcDNA3.1–6 × HisG-HMGCR, and confirmed by Western blot. **B**, **C** RT-qPCR (B) and Western blot (**C**) were used to assess the mRNA and protein levels of HMGCR and SREBP2 in the two paired stable cell lines. **D** Western blot analysis of HMGCR in shNUCB2-BT-549 and shNUCB2-MDA-MB-231 stable cell lines treated with exogenous Nesfatin-1 (0, 400, and 1000 pg/mL) for 48 h. **E** Representative IHC images of metastatic cancer tissues in nude mice were used to detect NUCB2, HMGCR, and SREBP2 expression. **F** Xena website analysis predicted consistent expression of NUCB2 and SREBP2. **G** Xena website analysis predicted consistent expression of NUCB2 and HMGCR. The statistical significance was verified using non-paired T-tests. *P < 0.05, **P < 0.01, ***P < 0.001. *EV* empty vector

We conducted further analysis of the IPA results to investigate the molecular mechanism underlying the up-regulation of cholesterol biosynthesis in metastasis induced by NUCB2. Through this analysis, we identified HMGCR as a differentially expressed protein which is a critical rate-limiting enzyme for cholesterol synthesis. To determine whether HMGCR also upregulates cholesterol synthesis in breast cancer, we transiently transfected pcDNA3.1–6 × HisG-HMGCR plasmid into BT-549 and MDA-MB-231 cells and observed that overexpression of HMGCR increased cholesterol synthesis (Fig. 5A).

As HMGCR is known to be a downstream target gene of the nuclear transcriptional factor SREBP2, which recognizes the sterol regulatory element (SRE) in the HMGCR gene promoter [37], we measured the expression of SREBP2 and HMGCR by RT-qPCR and Western blot in stable cell lines. We found that the knockdown of NUCB2 decreased the expression of SREBP2 and HMGCR compared to the control cell lines (Fig. 5B, C). In contrast, the exogenous Nesfatin-1 rescued HMGCR expression in a dose-dependent manner in shNUCB2-BT-549 and shNUCB2-MDA-MB-231 cell lines (Fig. 5D). To confirm the role of NUCB2-dependent HMGCR and SREBP2 expression in tumor metastasis in vivo, we performed immunohistochemical staining on metastatic lung tissues of nude mice. The results showed that NUCB2 depletion decreased the expression of SREBP2 and HMGCR compared to the control (Fig. 5E). These results provide evidence that NUCB2/Nesfatin-1 upregulates SREBP2 and HMGCR expression in breast cancer both in vitro and in vivo.

We also analyzed the expression of NUCB2 and SREBP2/HMGCR in breast cancer patients in the TCGA database and found a positive correlation between them (Fig. 5F, G). In addition, SREBP2/HMGCR expression was higher in breast cancer than in normal tissues and was related to poor prognosis (Additional file 1: Fig. S4A–D).

**Fig. 6:**
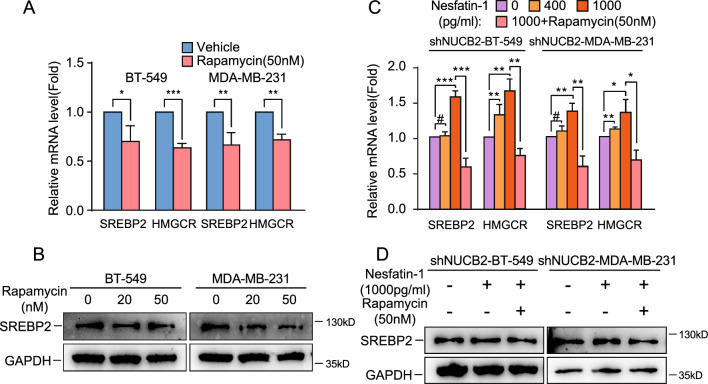
NUCB2/Nesfatin-1 upregulates SREBP2 and HMGCR expression via the mTORC1 pathway. **A** The mRNA expression levels of HMGCR and SREBP2 were measured by RT-qPCR in NUCB2 silencing and control cell lines treated with 50 nM rapamycin for 24 h. **B** Western blot analysis of SREBP2 expression in breast cancer cells treated with rapamycin (0, 20, or 50 nM) for 24 h. **C** RT-qPCR analysis of HMGCR and SREBP2 mRNA expression in shNUCB2-BT-549 and shNUCB2-MDA-MB-231 stable cell lines treated with exogenous Nesfatin-1 and rapamycin. The NUCB2 knockdown cell lines were starved for 24 h, followed by incubation with exogenous Nesfatin-1 (0, 400, and 1000 pg/mL) for 24 h. The RNA was extracted and used as templates for RT-qPCR. Another group of cells was starved for 24 h, treated with 50 nM rapamycin for 1 h, followed by incubation with 1000 pg/mL Nesfatin-1 for 24 h, and then the RNA was extracted and used as templates for RT-qPCR. **D** Western blot analysis of SREBP2 protein expression in NUCB2 knockdown cell lines treated with Nesfatin-1 combined with or without rapamycin. The NUCB2 knockdown cell lines were starved for 24 h, followed by incubation with 1000 pg/mL Nesfatin-1 for 24 h. Another group of cells was starved for 24 h, treated with 50 nM rapamycin for 1 h, followed by incubation with 1,000 pg/mL Nesfatin-1 for 24 h, and then the lysates were collected and analyzed by Western blot. The statistical significance was verified using a non-paired *t-*test. *P < 0.05, **P < 0.01, ***P < 0.001

We then investigated whether NUCB2 controls the SREBP2/HMGCR axis via the mammalian target of rapamycin (mTOR) signaling pathway, which is known to be a significant pathway regulating cholesterol synthesis [38, 39]. We verified this pathway in breast cancer cells by treating them with 50 nM rapamycin, a mTORC1 inhibitor, and found that the mRNA and protein levels of SREBP2 were decreased by rapamycin in BT-549 and MDA-MB-231 cells (Fig. 6A). We also treated NUCB2 knockdown the breast cancer cell lines with purified Nesfatin-1 and found that the expression of SREBP2 and HMGCR was upregulated in a dose-dependent manner (Fig. 6B). However, this effect was inhibited by rapamycin (Fig. 6C, D), indicating that NUCB2/Nesfatin-1 promotes the synthesis of SREBP2 and HMGCR by activating the mTORC1 signaling pathway, thus promoting cholesterol synthesis in breast cancer cells.

## Discussion

**Fig. 7:**
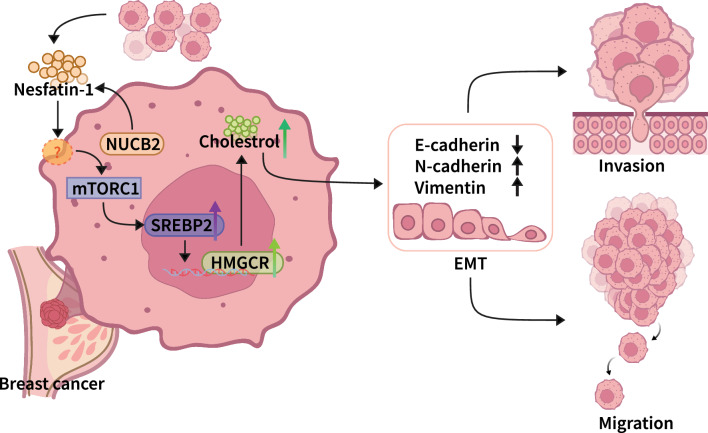
A schematic illustrating the role of NUCB2/Nesfatin-1 in regulating breast cancer invasion and metastasis via the mTORC1 signal pathway

In our previous study, Suzuki and I found that NUCB2 is highly expressed in breast cancer tissues and metastatic lymph nodes and is associated with poor prognosis [21, 24]. In this current study, we investigated the molecular mechanism underlying the role of NUCB2 in breast cancer. We first found that NUCB2 overexpression is potentially caused by acetylation in breast cancer. Subsequently, we generated NUCB2 knockdown breast cancer cell lines and found that these cells exhibited reduced invasion and metastasis. Nesfatin-1 could rescue the impaired invasion and metastasis by NUCB2 depletion. We also established a metastatic mouse model and confirmed that NUCB2 knockdown inhibited metastasis in vivo. Using IPA software, we found that cholesterol synthesis could be the primary pathway related to NUCB2, and we demonstrated that NUCB2/Nesfatin-1 upregulates cholesterol biosynthesis, promoting tumor invasion and metastasis. At the molecular mechanism level, the transcriptional activation of SREBP2 via the mTORC1 pathway plays a critical role in NUCB2/Nesfatin-1-induced cholesterol synthesis. To our knowledge, this is the first report to reveal that high NUCB2/Nesfatin-1 might promote breast cancer progression via upregulation of cholesterol synthesis depending on the mTORC1/SREBPS/HMGCR axis (Fig. 7). Our study defines the function of NUCB2/Nesfatin-1 in breast cancer and sheds light on potential therapeutic targets for this disease.

Cholesterol plays a crucial role as a structural component of cell membranes, particularly in lipid rafts that facilitate signal transduction [6]. Thus, cholesterol is likely to affect cellular architecture and signal transduction pathways. On the one hand, changes in membrane cholesterol content can modulate lipid raft activity and indirectly affect various signaling pathways, including the Wnt, hedgehog, and mTOR pathways, via low-density lipoprotein (LDL) receptor-related protein 5/6 receptor [40–42]. On the other hand, increased cholesterol in the breast cancer cell membranes can reduce membrane fluidity, leading to a more aggressive tumor phenotype characterized by enhanced cell motility, migration, and metastasis formation [43]. Kim et al. identified that the cholesterol synthesis pathway is significantly upregulated in a metastatic model of breast cancer, and pharmacological inhibition of this pathway reduced the breast cancer cell invasion [44]. Liu et al. demonstrated that high cholesterol stress can select for ferroptosis-resistant breast cancer cells with significantly increased tumorigenic and metastatic capacity [45]. Santos revealed that LDL-cholesterol induces epithelial-mesenchymal transition (EMT) by upregulating mesenchymal markers Slug, vimentin, and β-catenin and downregulating adhesion molecules cadherin-related family member 3(CDHR), CD226, Claudin, and Ocludin, thereby promoting migration and invasion of breast cancer cells [46].

Tumor metastasis is a complex process that involves the dissemination and distal colonization [47]. One of the most crucial steps of the metastatic cascade is the epithelial-mesenchymal transition (EMT), which is accompanied by multiple gene expression changes. Important hallmarks of EMT include the loss of epithelial markers, such as E-cadherin, β-catenin, Claudi-3, and the gain of mesenchymal markers, including N-cadherin, and vimentin [48]. In our study, we observed that NUCB2 downregulation impaired cell migration and invasion. We also examined EMT-related markers in stable cell lines and found that N-cadherin and Vimentin were decreased, while E-cadherin was increased in the shNUCB2-BT-549 and shNUCB2-MDA-MB-231 cell lines compared to the corresponding control cells. Therefore, our results suggest that NUCB2 promotes migration and invasion in breast cancer cells in vitro, likely through EMT (Fig. 7).

To investigate the signaling pathway underlying NUCB2-mediated cell invasion and metastasis, we used shNUCB2-MDA-MB-231 and scrambled MDA-MB-231 stable cell lines for gene expression analysis via Path Array strategy, followed by an analysis of the results using IPA software. Additional file 2: Table S3 shows a portion of the results, which indicate that five genes (FDFT1, DHCR7, ACAT2, HMGCR, and HMGCS1) involved in cholesterol biosynthesis were significantly downregulated in the shNUCB2-MDA-MB-231 cell line. This suggests that NUCB2/Nesfatin-1 potentially plays a crucial role in cholesterol biosynthesis, which was confirmed by IPA analysis. Our findings suggest that NUCB2/Nesfatin-1 promotes cholesterol synthesis in breast cancer cells, thereby enhancing cell invasion and metastasis. Furthermore, we found that HMGCR, a critical rate-limiting enzyme for cholesterol biosynthesis, was significantly downregulated in shNUCB2-BT-549 and shNUCB2-MDA-MB-231 cell lines compared to the corresponding control cells. This was confirmed by qPCR analysis. Taken together, our data indicate that NUCB2/Nesfatin-1 regulates cholesterol biosynthesis via the HMGCR/SREBP2 axis in breast cancer. However, as shown in Fig. 4A, there are still other signaling pathways involving NUCB2-related pathways such as cyclins and cell cycle regulation, Aryl Hydrocarbon receptor signaling, etc. Although our data indicate that NUCB2/Nesfatin-1 regulates cholesterol biosynthesis via the HMGCR/SREBP2 axis, further investigation is necessary to fully understand the role of NUCB2/Nesfatin-1 in breast cancer progression.

We analyzed the serum concentration of Nesfatin-1 in breast cancer patients and healthy controls and found that Nesfatin-1 concentration was higher in patients, corresponding to overexpression of NUCB2 in breast cancer tissues. We also detected the concentration of Nesfatin-1 in the cultured supernatant of the stable cell lines and found lower content of Nesfatin-1 in the supernatant of the shNUCB2 cells than in the control cells. Furthermore, we used the antibody-blocking experiment and found that the Nesfatin-1 antibody directly inhibits migration in breast cancer. On the other hand, Nesfatin-1 rescues metastasis impaired by NUCB2 depletion. In all, till now we first reported that NUCB2 promotes metastasis via the secreted fragment, Nesfatin-1 in breast cancer. Wang et al. also reported that the average level of nesfatin-1 in gastric cancer patients significantly increased compared with normal gastric tissue, and mediated the proliferation of gastric cancer cells [49]. These findings suggest that Nesfatin-1 is a potential diagnostic biomarker for breast cancer and gastric cancer. However, Kan et al. found that there was no difference in serum Nesfatin-1 levels between tumor patients and normal individuals, but NUCB2 promotes colorectal cancer EMT through the LKB1/AMPK/mTORC1/ZEB1 pathway [27]. Xu et al. reported that exogenous Nesfatin-1 dose-dependently inhibit the proliferation in ovarian cancer [50]. In all, these findings suggest that NUCB2 and Nesfatin-1 may independently play different roles in different tumors.

However, to date, specific receptors for NUCB2/Nesfatin-1 have not been identified. Recent findings suggest that NUCB2 exerts neuronal effects through G protein-coupled receptors (GPCRs) 3, 6, and 12, potential receptors for NUCB2 [51]. We found a new pattern of action for NUCB2 in breast cancer metastasis, and the further mechanism by NUCB2 activates mTORC1/SREBP2/HMGCR is not clear. In the future, Immunoprecipitation-Mass Spectrometry (IP-MS) can be used to scan the receptor molecules or extracellular binding proteins that bind to Nesfatin-1 and use it as a starting point to deeply explore the signal pathway of NUCB2/Nesfatin-1 activating mTORC1. This exploration can provide a potential target for the treatment or diagnosis of breast cancer with Nesfatin-1.

Post-transcriptional modifications (PTMs) refer to chemical modifications that occur on a protein after its translation, which regulates protein stability, activity, cellular localization, and interaction with other macromolecules [29, 30]. Each type of PTM is mainly catalyzed by three enzymes: writers, which add the modifications to substrates; erasers, which remove the modification from substrates; and readers, which recognize and bind to the modified substrates to perform the corresponding biological functions [31]. To preliminarily explore the mechanism of high expression of NUCB2 in breast cancer, we analyzed the potential acetylation sites in NUCB2 and the acetyltransferases involved using the CUCKOO database. We found that lysine at multiple locations of NUCB2 may be acetylated, and the acetyltransferases involved are mainly CREBBP, which has been reported to be activated in breast cancer and to elevate acetylation of many substrates [52]. Therefore, we treated breast cancer cell lines with different concentrations of the CREBBP inhibitor C646 and found that the expression level of NUCB2 decreased in a dose-dependent manner with increasing concentrations of C646, suggesting that NUCB2 may be overexpressed in breast cancer cells potentially through protein acetylation. In order to further explain the PTM mechanism of NUCB2, CREBBP family members should be screened to identify the specific acetyltransferase, and the exact lysine site should be confirmed. It is necessary to determine the eraser, writer, and reader so as to clearly clarify the molecular mechanism of high expression of NUCB2 in breast cancer.

In conclusion, our study demonstrates that NUCB2/Nesfatin-1 upregulates cholesterol synthesis, promoting breast cancer metastasis. This is achieved by enhancing the expression of the limiting-enzyme HMGCR via the mTORC1/SREBP2 pathway. In the future, we can screen corresponding antibodies and inhibitors targeting NUCB2/mTORC1/SREBP2/Cholesterol pathway, which inhibit tumor metastasis in vivo, resulting in laying a foundation for the development of therapeutic drugs related to translational medicine.

### Supplementary Information

**Additional file 1.** **Figure S1.** (**A**) The schematic diagram depicts a pattern of secretory Nesfatin-1, 2, and 3 that NUCB2 splits to form. (**B**) The potential acetylated lysine residue of NUCB2 and the involved acetyltransferases were predicted by using the CUCKOO database. (**C**) The possibility of acetyltransferases involved in NUCB2 acetylation is predicted by the CUCKOO database. (**D**) Cell viability after being treated by C646 with different concentrations (0-80μM) for 24h was detected by CCK-8 reagent. **Figure S2.** Confirmation of NUCB2/Nesfatin-1 knockdown and scrambled stable cell lines in BT-549 and MDA-MB-231 determined by RT-qPCR (**A**), Western blot (**B**), and ELISA for detection of the Nesfatin-1 concentrations in the culture supernatant (**C**). 2×105 cell lines were seeded respectively in a six-well plate containing 2 mL medium nd cultured overnight. The supernatants were collected and detected the Nesfatin-1 concentration by ELISA. The unpaired T-test was used to verify the statistical significance. **P < 0.01, ***P < 0.001. **Figure S3.** (**A**) Cell activity after being treated with exogenous Nesfatin-1 with different concentrations (0, 10, 100, 1,000, and 10,000 pg/mL) measured by CCK-8 reagent. (**B**) Colony formation assay results in shNUCB2-BT-549 and shNUCB2-MDA-MB231 stable cell lines treated with 1000 pg/mL Nesfatin-1. The unpaired T-test was used to verify the statistical significance. *P < 0.05, **P < 0.01. **Figure S4.** (**A**) TCGA data showed SREBP2 mRNA expression in primary breast cancer tissues (*n* = 1097) and normal tissues (*n* = 114). (*B*) TCGA data showed HMGCR mRNA expression in primary breast cancer tissues (*n* = 1097) and normal tissues (*n* = 114). (**C**) Kaplan-Meier survival curve at univariate SREBP2 level based on TCGA data. (**D**) Kaplan-Meier survival curve at univariate HMGCR level based on TCGA data. The unpaired T-test was used to verify the statistical significance. *P < 0.05, **P < 0.01, ***P < 0.001.

**Additional file 2: Table S1**. Primary antibodies and Second antibodies. **Table S2**. Primers for RT-qPCR. **Table S3**. IPA results of FDFT1, DHCR7, ACAT2, HMGCR, HMGCS15 genes.

## Data Availability

The original contributions presented in the study are included in the article/Additional files. Further inquiries can be directed to the corresponding author.

## Abbreviations

ELISA: Enzyme-linked immuno-sorbent assay
HMGCR: 3-Hydroxy-3-methyl glutaryl-CoA reductase
IPA: Ingenuity pathway analysis
mTORC1: Mammalian target of rapamycin complex 1
NUCB2: Nucleobindin-2
SREBP2: Sterol regulatory element-binding protein 2
