# Correction: NUCB2/Nesfatin-1 drives breast cancer metastasis through the up-regulation of cholesterol synthesis via the mTORC1 pathway

**DOI:** 10.1186/s12967-023-04385-z

**Published:** 2023-08-03

**Authors:** Siyi Ning, Caiying Liu, Kangtao Wang, Yubo Cai, Zhicheng Ning, Ming Li, Liang Zeng

**Affiliations:** 1 Department of Immunology, College of Basic Medical Sciences, Central South University, Changsha, 410028 China; 2 Department of General Surgery, The Xiangya Hospital, Central South University, Changsha, 410028 China; 3 Hunan Normal University School of Medicine, Changsha, 410031 China; 4 Department of Pathology, Guangzhou Women and Children’s Medical Center, Guangzhou Medical University, Guangdong Provincial Clinical Research Center for Child Health, National Children’s Medical Center for South Central Region, Guangzhou, 510623 China

**Correction: Journal of Translational Medicine (2023) 21:362** **https://doi.org/10.1186/s12967-023-04236-x**

Following publication of the original article [1], we have been notified that Dr. Ming Li should also be marked as the corresponding author. The authors’ group is corrected in this erratum.
