# Supplementary figures and images for: NUCB2/Nesfatin-1 drives breast cancer metastasis through the up-regulation of cholesterol synthesis via the mTORC1 pathway
Source: J Transl Med. 2023 Jun 5;21:362. doi: 10.1186/s12967-023-04236-x (PMC10243030; doi:10.1186/s12967-023-04236-x)

Figure.S1

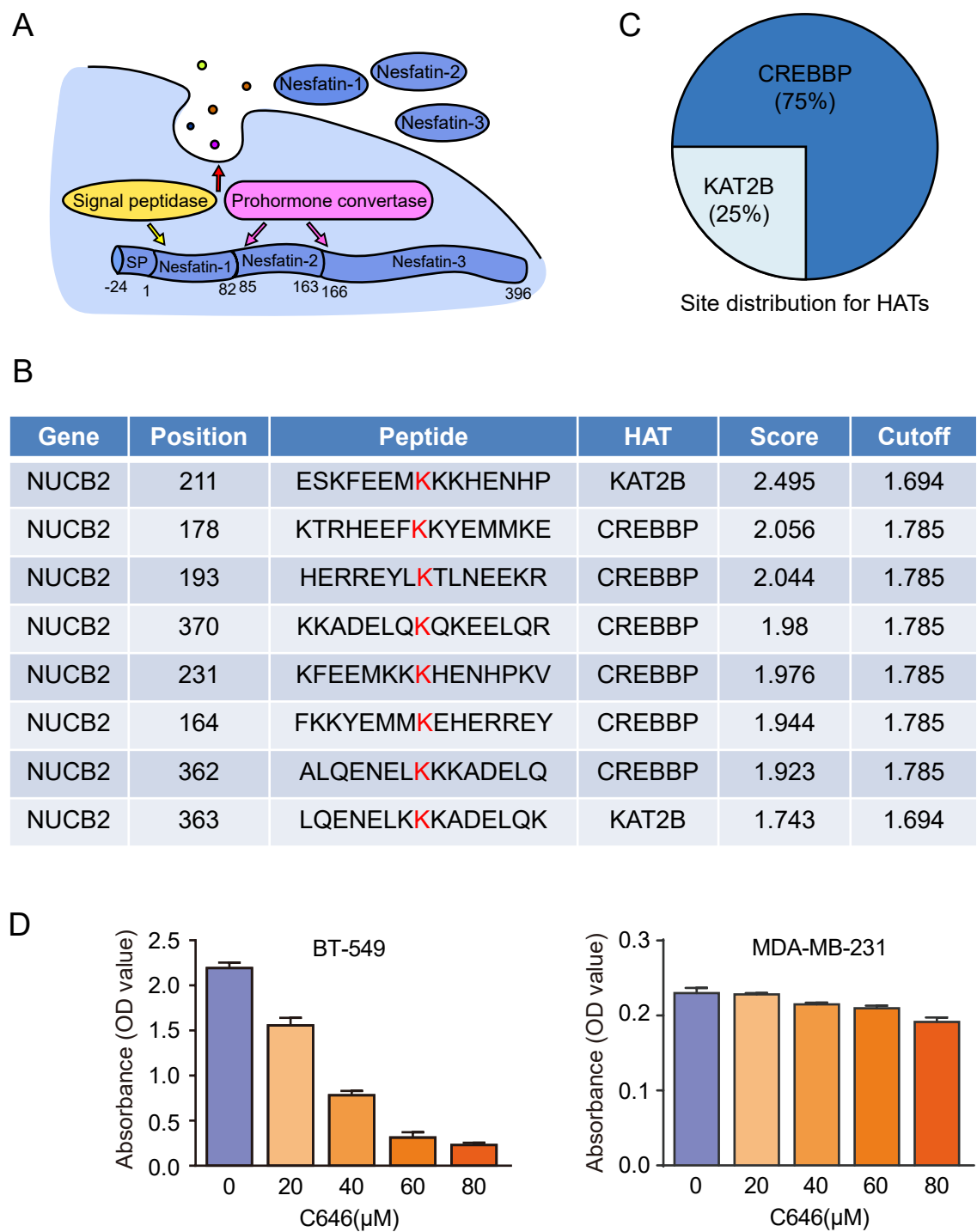

Figure.S2

A

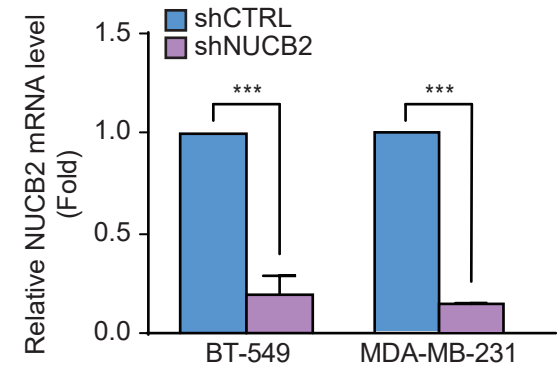

C

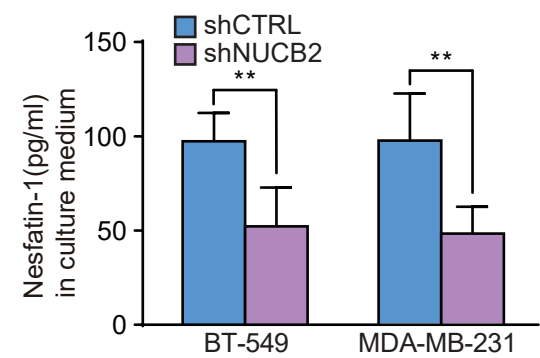

B

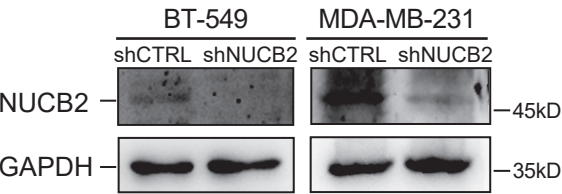

Ning et al., Fig S2

Figure.S3

A

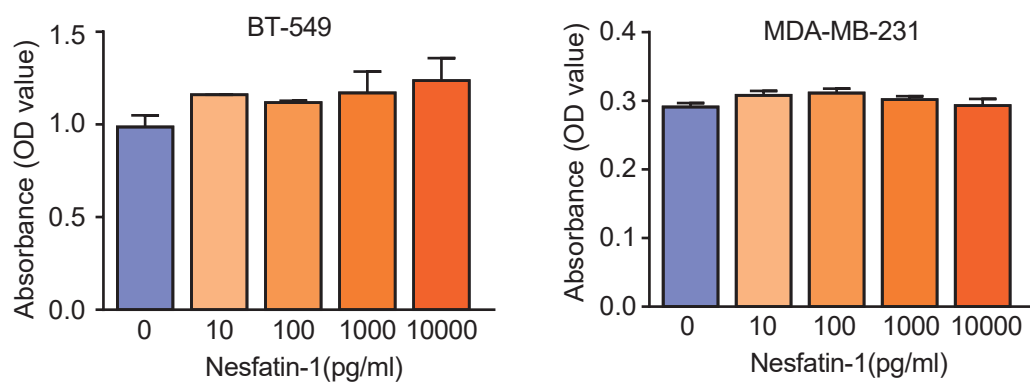

B

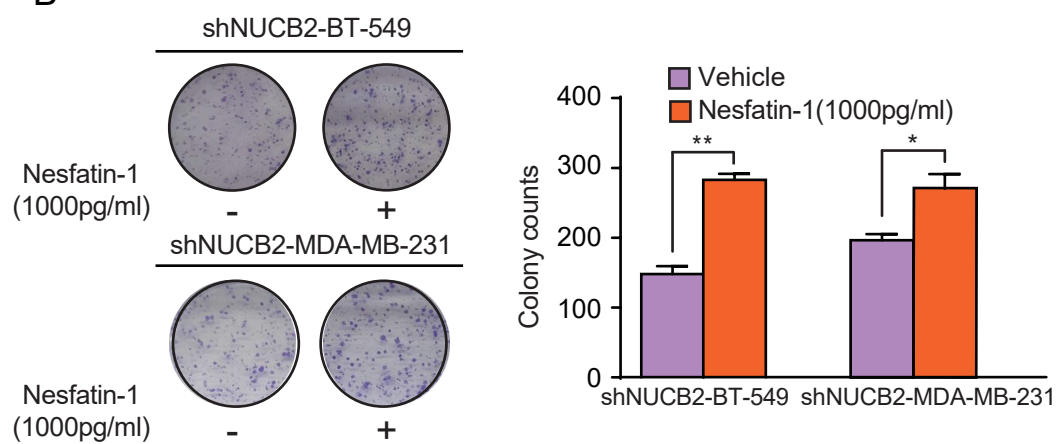

Figure.S4

A

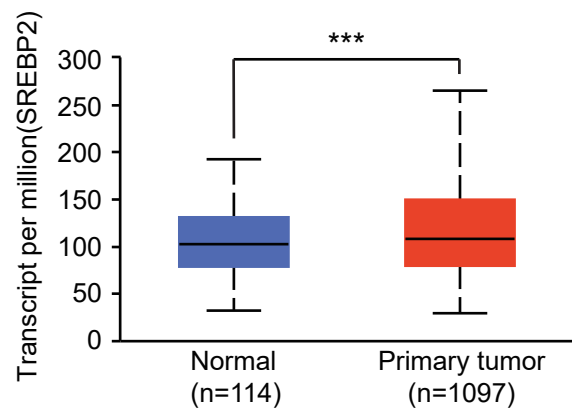

B

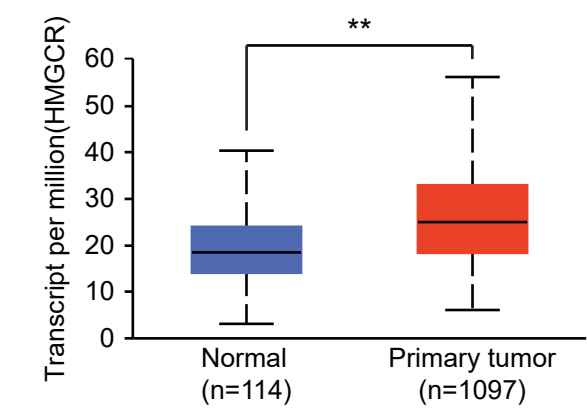

C

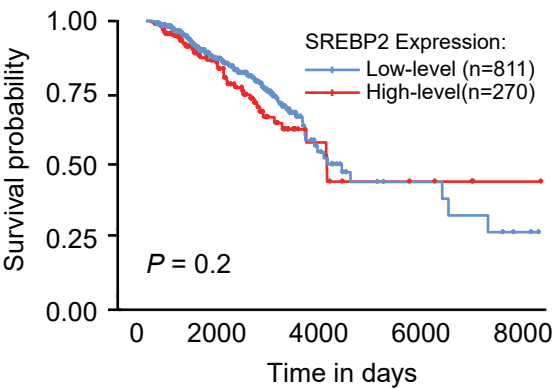

D

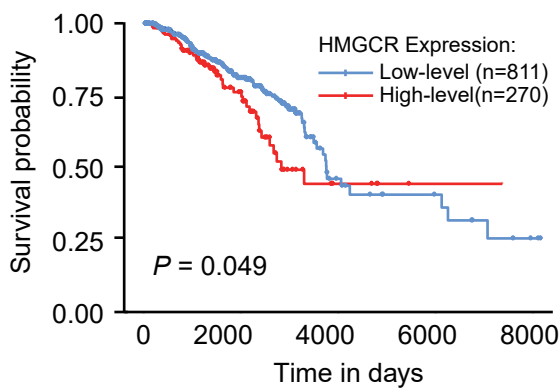

Supplement: Supplementary file 1 — Additional file 1. Figure S1. (A) The schematic diagram depicts a pattern of secretory Nesfatin-1, 2, and 3 that NUCB2 splits to form. (B) The potential acetylated lysine residue of NUCB2 and the involved acetyltransferases were predicted by using the CUCKOO database. (C) The possibility of acetyltransferases involved in NUCB2 acetylation is predicted by the CUCKOO database. (D) Cell viability after being treated by C646 with different concentrations (0-80μM) for 24h was detected by CCK-8 reagent. Figure S2. Confirmation of NUCB2/Nesfatin-1 knockdown and scrambled stable cell lines in BT-549 and MDA-MB-231 determined by RT-qPCR (A), Western blot (B), and ELISA for detection of the Nesfatin-1 concentrations in the culture supernatant (C). 2×10^5^ cell lines were seeded respectively in a six-well plate containing 2 mL medium nd cultured overnight. The supernatants were collected and detected the Nesfatin-1 concentration by ELISA. The unpaired T-test was used to verify the statistical significance. **P < 0.01, ***P < 0.001. Figure S3. (A) Cell activity after being treated with exogenous Nesfatin-1 with different concentrations (0, 10, 100, 1,000, and 10,000 pg/mL) measured by CCK-8 reagent. (B) Colony formation assay results in shNUCB2-BT-549 and shNUCB2-MDA-MB231 stable cell lines treated with 1000 pg/mL Nesfatin-1. The unpaired T-test was used to verify the statistical significance. *P < 0.05, **P < 0.01. Figure S4. (A) TCGA data showed SREBP2 mRNA expression in primary breast cancer tissues (n = 1097) and normal tissues (n = 114). (B) TCGA data showed HMGCR mRNA expression in primary breast cancer tissues (n = 1097) and normal tissues (n = 114). (C) Kaplan-Meier survival curve at univariate SREBP2 level based on TCGA data. (D) Kaplan-Meier survival curve at univariate HMGCR level based on TCGA data. The unpaired T-test was used to verify the statistical significance. *P < 0.05, **P < 0.01, ***P < 0.001. [file 12967_2023_4236_MOESM1_ESM.pdf]
