# Supplementary material for: NUCB2/Nesfatin-1 drives breast cancer metastasis through the up-regulation of cholesterol synthesis via the mTORC1 pathway
Source: J Transl Med. 2023 Jun 5;21:362. doi: 10.1186/s12967-023-04236-x (PMC10243030; doi:10.1186/s12967-023-04236-x)
Supplement: Supplementary file 2 — Additional file 2: Table S1. Primary antibodies and Second antibodies. Table S2. Primers for RT-qPCR. Table S3. IPA results of FDFT1, DHCR7, ACAT2, HMGCR, HMGCS15 genes. [file 12967_2023_4236_MOESM2_ESM.docx]

Table S1. Primary antibodies and Second antibodies

| **Method** | **Primary Antibody** | **Titer** | **Secondary Antibody** | **Titer** |
| --- | --- | --- | --- | --- |
| WB | anti-GAPDH | 1:10000 | anti-Rabbit IgG | 1:3000 |
| WB | anti-NUCB2 | 1:100 | anti-Rabbit IgG | 1:3000 |
| IHC | anti-NUCB2 | 1:100 | anti-Rabbit IgG | 1:200 |
| WB | anti-E-cadherin | 1:1000 | anti-Rabbit IgG | 1:3000 |
| WB | anti-N-cadherin | 1:1000 | anti-Rabbit IgG | 1:3000 |
| WB | anti-Vimentin | 1:1000 | anti-Rabbit IgG | 1:3000 |
| WB | anti-HisG | 1:1000 | anti-Rabbit IgG | 1:3000 |
| WB | anti-HMGCR | 1:100 | anti-Mouse IgG | 1:4000 |
| IHC | anti- HMGCR | 1:100 | anti-Mouse IgG | 1:150 |
| WB | anti-SREBP2 | 1:1000 | anti-Rabbit IgG | 1:3000 |
| IHC | anti-SREBP2 | 1:200 | anti-Rabbit IgG | 1:200 |

Table S2. Primers for RT-qPCR

| **Gene** | **Forward** | **Reverse** |
| --- | --- | --- |
| GAPDH | 5'- CATGGGTGTGAACCATGAGAA -3' | 5'- GGTCATGAGTCCTTCCACGAT -3' |
| NUCB2 | 5'-CCTGTGGAAAGTGCGAAGATAG-3' | 5'-GCCTCCCACTCTTTATTTCCTC-3' |
| HMGCR | 5'- TGATTGACCTTTCCAGAGCAAG -3' | 5'- CTAAAATTGCCATTCCACGAGC -3' |
| SREBP2 | 5'- CTCCATTGACTCTGAGCCAGGA -3' | 5'- GAATCCGTGAGCGGTCTACCAT -3' |

| **Symbol** | **Entrez Gene Name** | **FDR** | **P-value** | **Fold Change** | **logFC** | **Absolute FC** | **Pathway** |
| --- | --- | --- | --- | --- | --- | --- | --- |
| ACAT2 | acetyl-CoA acetyltransferase 2 | 6.86445E-12 | 4.30808E-15 | -1.733466553 | -0.79366 | 1.733466553 | Cholesterol_Bios |
| FDFT1 | farnesyl-diphosphate farnesyltransferase 1 | 1.30689E-11 | 1.13998E-14 | -1.620414987 | -0.69636 | 1.620414987 | Cholesterol_Bios |
| DHCR7 | 7-dehydrocholesterol reductase | 2.97383E-11 | 4.39497E-14 | -1.627536615 | -0.70269 | 1.627536615 | Cholesterol_Bios |
| HMGCR | 3-hydroxy-3-methylglutaryl-CoA reductase | 4.38546E-09 | 5.08729E-11 | -1.522634846 | -0.60657 | 1.522634846 | Cholesterol_Bios |
| HMGCS1 | 3-hydroxy-3-methylglutaryl-CoA synthase 1 | 6.86514E-09 | 9.32587E-11 | -1.760017776 | -0.81559 | 1.760017776 | Cholesterol_Bios |

Table S3. IPA results of FDFT1, DHCR7, ACAT2, HMGCR, HMGCS15 genes
